# Supplementary material for: A first-takes-all model of centriole copy number control based on cartwheel elongation
Source: PLoS Comput Biol. 2021 May 10;17(5):e1008359. doi: 10.1371/journal.pcbi.1008359 (PMC8136855; doi:10.1371/journal.pcbi.1008359)
Supplement: S1 Table — (PDF) [file pcbi.1008359.s001.pdf]

Table S1. Parameters across all models

Parameter	Definition	Units	Range
σ	SAS-6 influx rate. Simplification of SAS-6 expression and degradation dynamics, as well as centrosomal recruitment.	molecules t^{-1}	[0,∞[
k_{on}	Oligomerisation rate. Rate at which two SAS-6 oligomers of the same or different sizes combine.	molecules$^{-1}t^{-1}$	[0,∞[
k_{off}	Dissociation rate. Rate at which a SAS-6 oligomer dissociates into any combination of two of its constituents.	t^{-1}	[0,∞[
k_s	Stacking rate. Rate at which a SAS-6 oligomer combines with a cartwheel by stacking on top of it.	molecules$^{-1}t^{-1}$	[0,∞[
k_u	Stack dissociation rate. Rate at which a SAS-6 oligomer dissociates from the top of a cartwheel stack.	t^{-1}	[0,∞[
r	Ring size. Number of SAS-6 oligomers in a complete ring, necessary to form a new, distinct cartwheel.	molecules	$\mathbb{N}$
h	Maximum cartwheel length. Maximum number of stacked rings in a cartwheel, at which further stacking reactions are blocked.	rings	$\mathbb{N}$
ρ	Mean feedback time. Mean time after the first cartwheel formation event at which further synthesis and elongation are prevented (i.e. the simulation is stopped).	t	[0,∞[
α	Shape parameter of the gamma-distribution of feedback times. When α=1 feedback time simplifies to an exponential distribution.	nondimensional	[1,∞[
