# Supplementary material for: A first-takes-all model of centriole copy number control based on cartwheel elongation
Source: PLoS Comput Biol. 2021 May 10;17(5):e1008359. doi: 10.1371/journal.pcbi.1008359 (PMC8136855; doi:10.1371/journal.pcbi.1008359)
Supplement: S1 Fig — (A-C) Relative frequency of simulations with the indicated number of cartwheels, at the stopping time. We performed 1000 simulations with no SAS-6 molecules initially present and stopped them at time t = 100; time t is measured in arbitrary units (a.u.). For all simulations, kon = koff = 1. Note that ks = 0 represents absence of stacking. See also Models and methods section for default initial and stopping conditions, and parameter values for the simulations. (PDF) [file pcbi.1008359.s002.pdf]

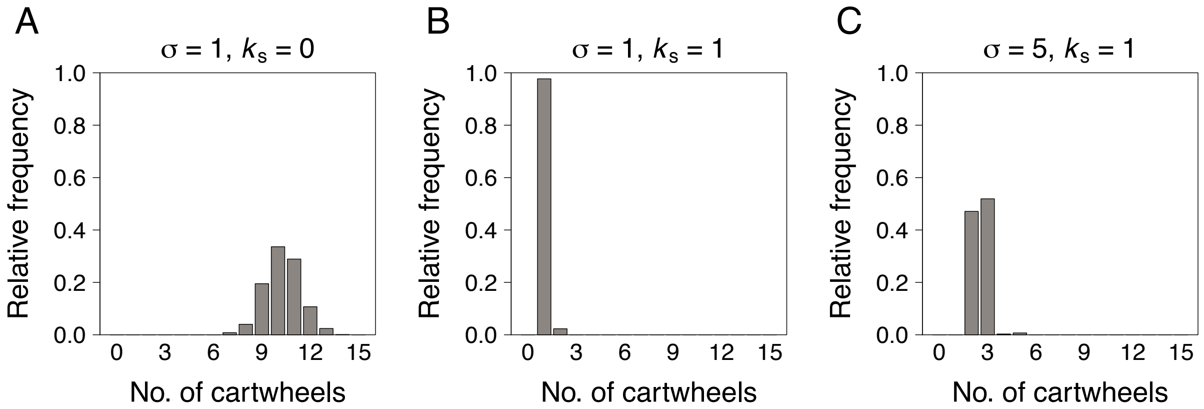

S1 Fig Final cartwheel number distributions. (A-C) Relative frequency of simulations with the indicated number of cartwheels, at the stopping time. We performed 1000 simulations with no SAS-6 molecules initially present and stopped them at time $t = 100$; time t is measured in arbitrary units (a.u.). For all simulations, $k_{on} = k_{off} = 1$. Note that $k_s = 0$ represents absence of stacking. See also Models and Methods section for default initial and stopping conditions, and parameter values for the simulations.
