# Supplementary material for: A first-takes-all model of centriole copy number control based on cartwheel elongation
Source: PLoS Comput Biol. 2021 May 10;17(5):e1008359. doi: 10.1371/journal.pcbi.1008359 (PMC8136855; doi:10.1371/journal.pcbi.1008359)
Supplement: S2 Fig — (A-C) Relative frequency of simulations where the first (black) and second (red) cartwheels contained the indicated number of stacked rings, at the stopping time. We used default simulation settings as indicated in S1 Fig and described in section Models and methods. (PDF) [file pcbi.1008359.s003.pdf]

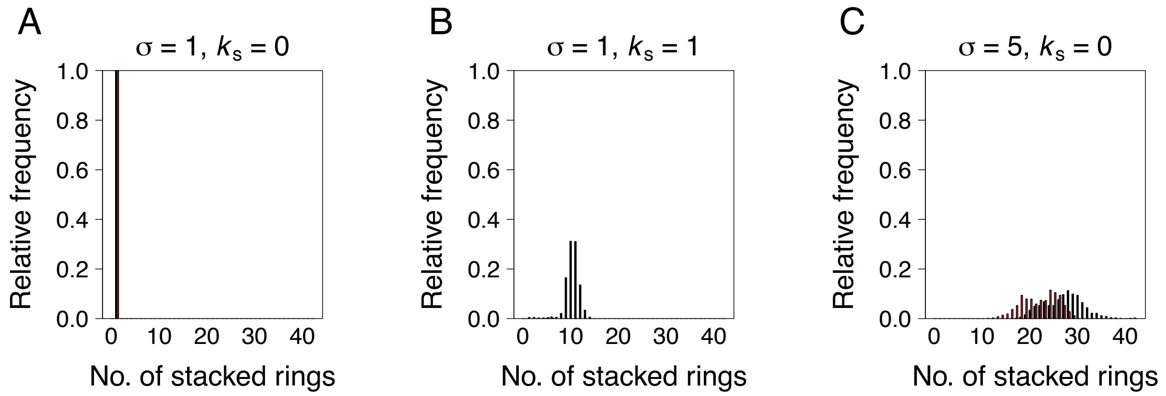

S2 Fig Final cartwheel length distributions. (A-C) Relative frequency of simulations where the first (black) and second (red) cartwheels contained the indicated number of stacked rings, at the stopping time. We used default simulation settings as indicated in S1 Fig and described in section Models and Methods.
