# Supplementary material for: A first-takes-all model of centriole copy number control based on cartwheel elongation
Source: PLoS Comput Biol. 2021 May 10;17(5):e1008359. doi: 10.1371/journal.pcbi.1008359 (PMC8136855; doi:10.1371/journal.pcbi.1008359)
Supplement: S4 Fig — (A-C) We used default simulation settings as indicated in S1 Fig and described in section Models and methods. (PDF) [file pcbi.1008359.s005.pdf]

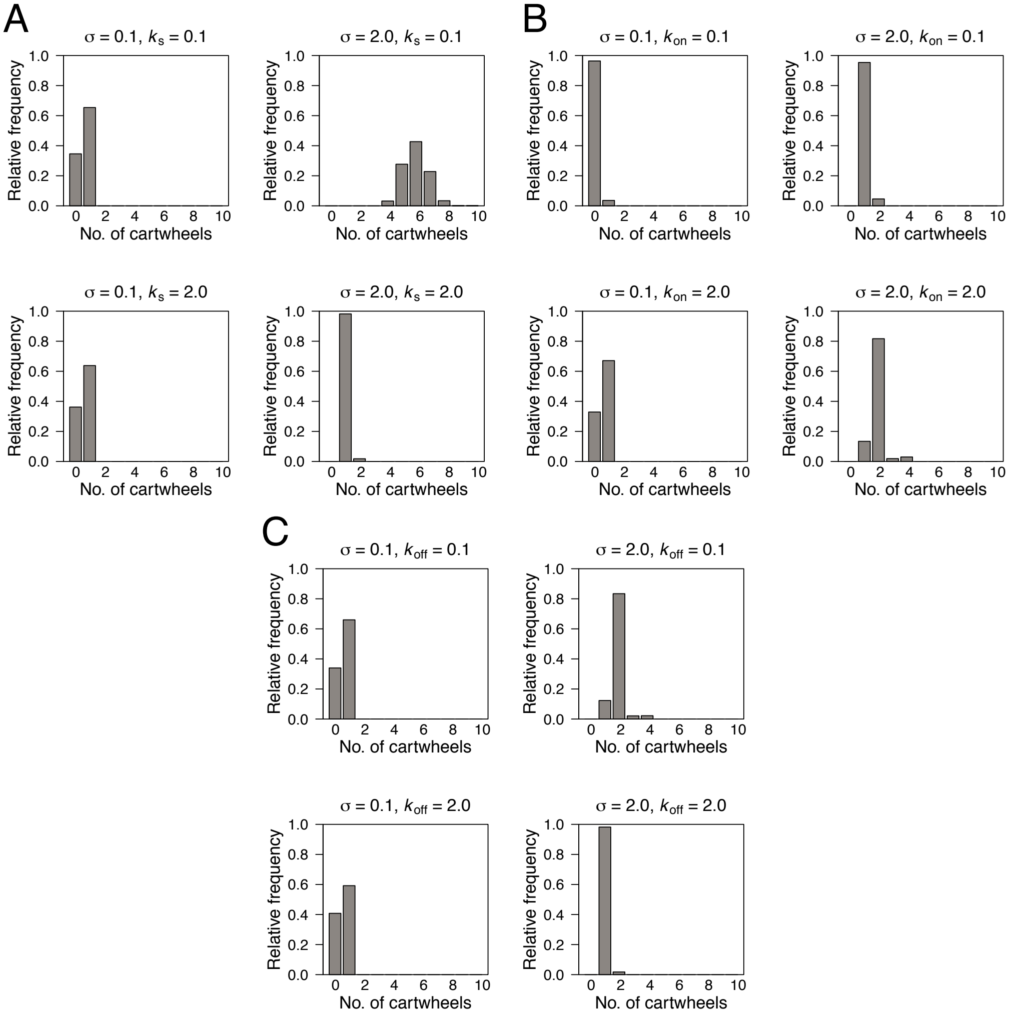

S4 Fig Final distribution of cartwheel numbers as a function of influx and different reaction rate parameters. (A-C) We used default simulation settings as indicated in S1 Fig and described in section Models and Methods
