# Supplementary material for: A first-takes-all model of centriole copy number control based on cartwheel elongation
Source: PLoS Comput Biol. 2021 May 10;17(5):e1008359. doi: 10.1371/journal.pcbi.1008359 (PMC8136855; doi:10.1371/journal.pcbi.1008359)
Supplement: S6 Fig — Relative frequency of simulations containing a single cartwheel at time t = 100, under the model assuming reversible ring assembly and stacking. We varied the parameter values indicated in the x- and y-axes in steps of 0.1, yielding a total of 400 pairwise combinations of parameter values per plot. We used default simulation settings as indicated in S1 Fig and described in section Models and methods, and set ku = 1. Note that ks = 0 represents absence of stacking. (PDF) [file pcbi.1008359.s007.pdf]

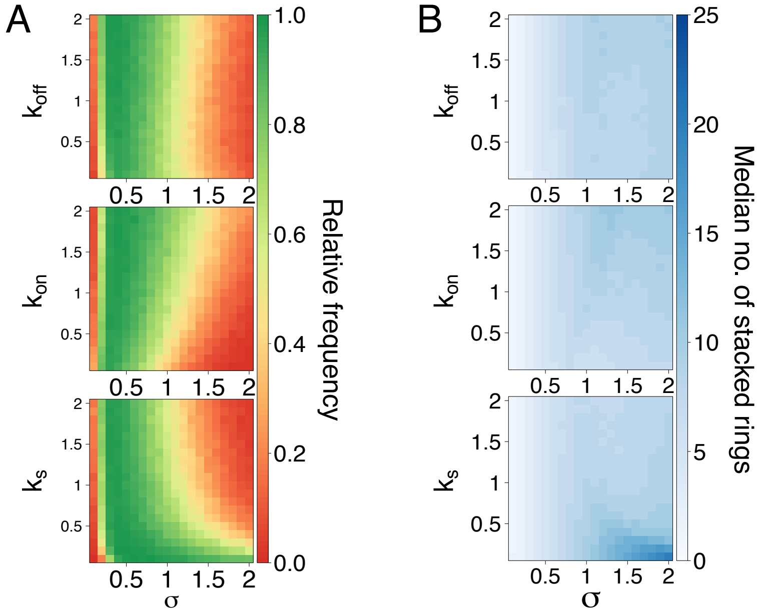

S6 Fig Probability of forming one and only one cartwheel as a function of influx and reaction parameters, assuming reversible ring formation and stacking. Relative frequency of simulations containing a single cartwheel at time $t = 100$, under the model assuming reversible ring assembly and stacking. We varied the parameter values indicated in the x- and y-axes in steps of 0.1, yielding a total of 400 pairwise combinations of parameter values per plot. We used default simulation settings as indicated in S1 Fig and described in section Models and Methods, and set $k_u = 1$. Note that $k_s = 0$ represents absence of stacking
