# Supplementary material for: A first-takes-all model of centriole copy number control based on cartwheel elongation
Source: PLoS Comput Biol. 2021 May 10;17(5):e1008359. doi: 10.1371/journal.pcbi.1008359 (PMC8136855; doi:10.1371/journal.pcbi.1008359)
Supplement: S7 Fig — Presence of the first (black) and second (red) cartwheels along time, under the model assuming reversible ring assembly and stacking, in a random sub-sample of 100 out of 1000 simulations. We used default simulation settings as indicated in S1 Fig and described in section Models and methods, and set ku = 1. A—ks = 1; B—ks = 5. (PDF) [file pcbi.1008359.s008.pdf]

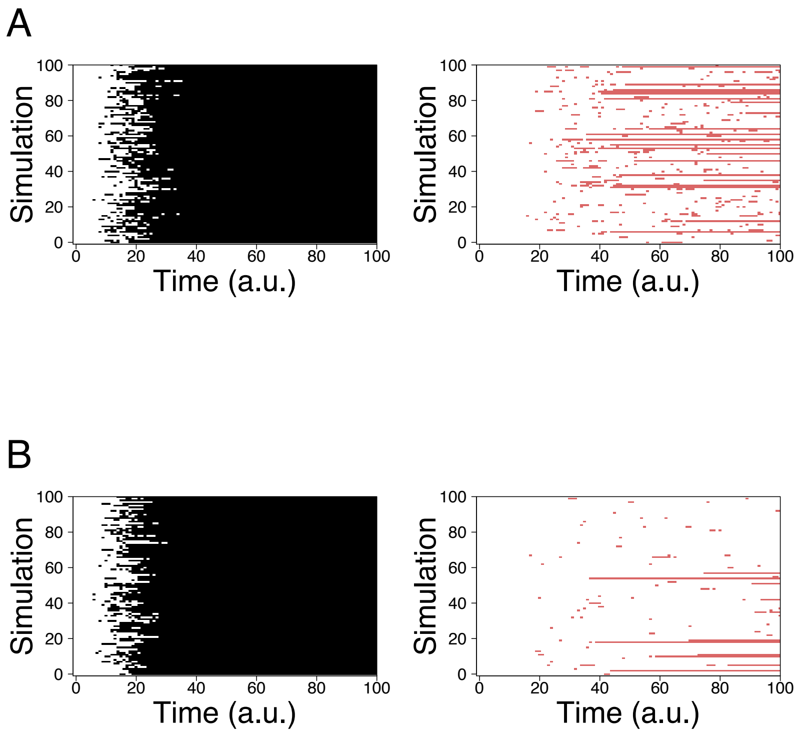

S7 Fig The first assembled cartwheel is stably maintained whereas the second one is dynamically assembled and disassembled. Presence of the first (black) and second (red) cartwheels along time, under the model assuming reversible ring assembly and stacking, in a random sub-sample of 100 out of 1000 simulations. We used default simulation settings as indicated in S1 Fig and described in section Models and Methods, and set $k_u = 1$. A - $k_s = 1$; B - $k_s = 5$.
