# Supplementary material for: A first-takes-all model of centriole copy number control based on cartwheel elongation
Source: PLoS Comput Biol. 2021 May 10;17(5):e1008359. doi: 10.1371/journal.pcbi.1008359 (PMC8136855; doi:10.1371/journal.pcbi.1008359)
Supplement: S8 Fig — (A) Assembly of supernumerary cartwheels is progressively delayed as the maximum cartwheel length h increases. (B) The distribution of feedback times simplifies to an exponential distribution for α = 1, with mean ρ. Otherwise, it is gamma-distributed. (C) Assembly times of the second cartwheel as a function of feedback parameters. We used default simulation settings as indicated in S1 Fig and described in section Models and methods. (PDF) [file pcbi.1008359.s009.pdf]

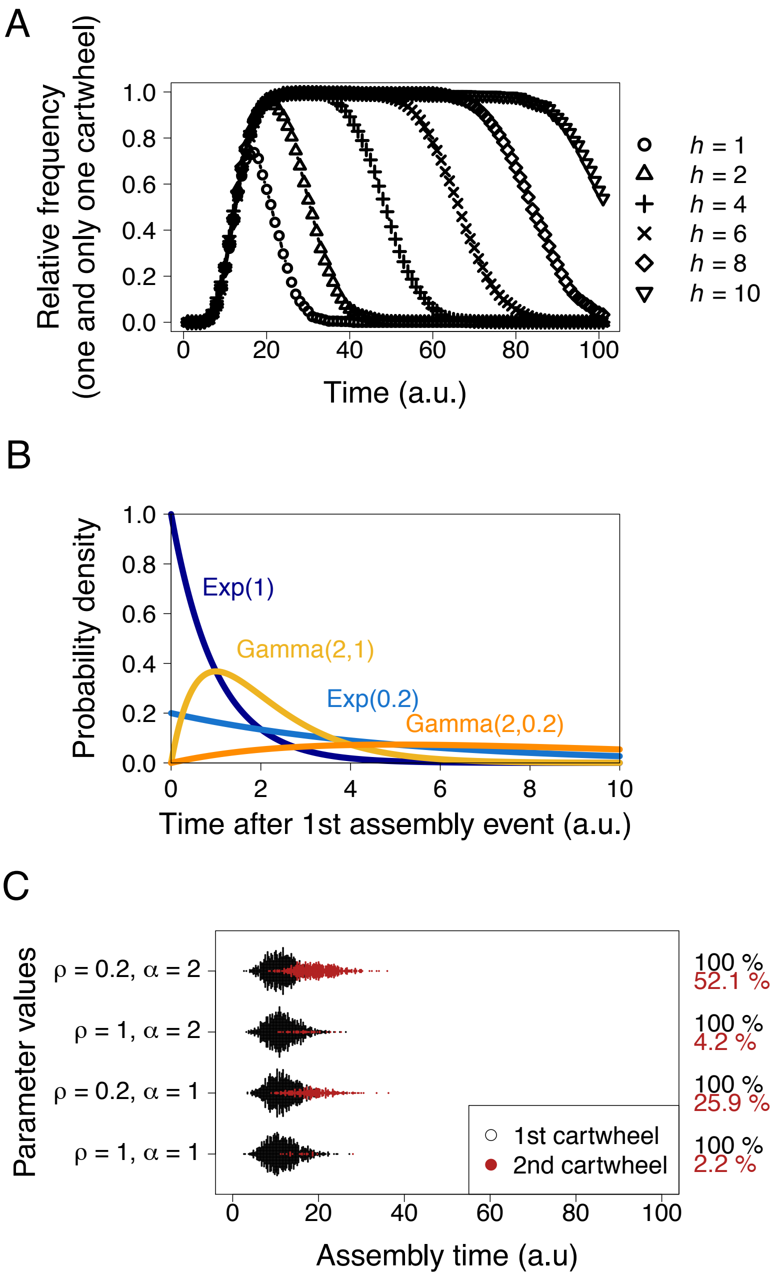


**S8 Fig** Additional features of the limited stacking and feedback models. (A) Assembly of supernumerary cartwheels is progressively delayed as the maximum cartwheel length $h$ increases. (B) The distribution of feedback times simplifies to an exponential distribution for $\alpha = 1$, with mean $\rho$. Otherwise, it is gamma-distributed. (C) Assembly times of the second cartwheel as a function of feedback parameters. We used default simulation settings as indicated in S1 Fig and described in section Models and Methods.
