# Supplementary material for: A first-takes-all model of centriole copy number control based on cartwheel elongation
Source: PLoS Comput Biol. 2021 May 10;17(5):e1008359. doi: 10.1371/journal.pcbi.1008359 (PMC8136855; doi:10.1371/journal.pcbi.1008359)
Supplement: Attachment — Submitted filename: ReplyToTheReviewers.pdf [file pcbi.1008359.s010.pdf]

Reviewer's Responses to Questions

Comments to the Authors:

Please note here if the review is uploaded as an attachment.

Reviewer #1: In this manuscript, Dias Louro M.A. and colleagues proposed a new model to explain how centriole copy number is strictly limited to one. By focusing on the cartwheel assembly and stacking, they generated a mathematical model and used computer simulations for a plausible solution to centriole copy number control. The essence would be that the first cartwheel functions as a template and stacks layers of other cartwheels. Due to this, the formed intermediates of SAS-6 oligomers are incorporated into the first cartwheel pile. Due to the lack of intermediate cartwheel building blocks, second cartwheel pile is not formed at other sites. Based on the models with a certain range of parameters, the authors found the conditions in which only one cartwheel pile is formed within a given assembly time. To understand the principles of centriole copy number control, it is important in the field to raise and compare reasonable theories and models, and test them experimentally. However, this reviewer feels that this model could explain only a small part of the mechanisms of centriole copy number control. It may well explain the dynamics of cartwheel assembly after site selection of centriole duplication, but it would not fit the various observations related to centriole copy number control in the past studies. This reviewer hopes that the following comments will help improve their models in some way.

We thank the reviewer for his/her point and believe the importance and context of our model might have not been clear in our previous version. We tried to make these clearer in the introduction writing on L25-L39:

Several studies proposed that Plk4 and STIL have an essential role in controlling centriole copy numbers by accumulating the essential components in a local focus around the mother centriole [19–23]. Mathematical models describing these focusing dynamics based on different mechanisms have been put forward [14,23–25]. These models were formulated in terms of the local Plk4, STIL, and SAS-6 concentrations, which are continuous quantities untranslatable to discrete centriole numbers. To overcome this basic limitation [14] and [24] defined discrete spatial compartments around the mother centriole, each being able to harbour one and only one centriole by construction. This limitation notwithstanding, the focused accumulation explains naturally the prevention of ectopic centrioles by the maintenance of the key components concentrations below critical values elsewhere in the cell and around the mother centriole. However, these (qualitative or quantitative) proposals fail to explain how the formation of supernumerary centrioles is avoided at the foci, where Plk4, STIL and the other components accumulate at supercritical concentrations. This is the control 38 problem that is solved by the first-takes-all model proposed here.

-Major points

1)The authors should consider the fact that overexpression of Plk4 or STIL also induces centriole overduplication, which might contradict their models. Moreover, SAS-6 loading to centrioles and the cartwheel assembly require Plk4 and STIL. In particular, considering that Plk4 is upstream of the SAS-6 loading, the regulation for centriole copy number is more critically exerted on Plk4. How could the authors fit these observations to their models and simulation results? The authors should discuss this issue in the manuscript.

We thank the reviewer for calling our attention to the fact that our original manuscript left implicit some assumptions on SAS-6 influx. Although we mentioned in the second paragraph of the Discussion that we assumed that SAS-6 influx depends on Plk4 and STIL, the original description of the model did not make justice to the complexity of the regulation of SAS-6 levels, and its dependence on these key upstream components. We revised the manuscript, making explicit that the complexity of the control of SAS-6 activity by upstream regulators Plk4 and STIL is lumped in the influx parameter. We added the following text to clarify this:

L102-L111:

To explore these potential issues, we formulated a more realistic model describing the reaction system of cartwheel assembly.

We consider that the reaction system is contained in the focal volume around a pre-existing mother centriole. Fluorescence measurements of SAS-6 intensities at centrioles indicate that it accumulates during S-phase, peaking in G2 [12,30]. As mentioned above, cytosolic SAS-6 has been found preferentially in homodimer form [30]. In accordance with these observations we assume that SAS-6 expression, degradation, dimerisation and recruitment to the centrosome through the action of Plk4 and STIL, result in a constant influx of SAS-6 homodimers, C1, into the focal volume, at a rate σ.

L335-L352:

The dependence of cartwheel formation on the influx parameter in our model is in agreement with empirical observations of centriole under- and overproduction upon SAS-6 depletion and overexpression, respectively. However, the influx parameter represents in a single average quantity a complex process that includes recruitment via Plk4 and STIL, which is spatially regulated [19,22]. This implies that the influx parameter does not correspond to SAS-6 expression level directly; rather, it is best approximated by the balance between the rate of SAS-6 loading onto the centrosome, and subsequent turnover, both of which are more challenging to quantify. Furthermore, the levels of Plk4, STIL, and SAS-6 proteins are regulated by degradation, which in some cases can lead to antagonistic interactions, e.g. Plk4 can recruit SAS-6 but may also target it for degradation through FBXW5 [16,18]. In addition, since the kinetics of SAS-6 recruitment by Plk4 and STIL are poorly understood, it is possible that they are nonlinear. Altogether, there may be complex interactions between Plk4, STIL, and SAS-6, leading to behaviours that cannot be obtained by assuming that cartwheel number is directly proportional to the total abundance of these proteins in the cell.

2) In the models, the authors consider a focal volume around a pre-existing that is large enough to allow for mass action dynamics. What is the basis for this premise? Based on the previous observations, it is likely that SAS-6 dimers are formed in the cytoplasm and targeted into Plk4 around the pre-existing centriole. Although it is possible that SAS-6 influx affects Plk4 dynamics, the site for SAS-6 loading around the pre-existing centriole is basically limited by pre-existing Plk4. Even in this scenario, are their models still feasible?

We thank the reviewer for this question which gave us another opportunity to clarify our model. Very briefly, this is a simplifying assumption that is essential to allow us to derive the analytical solution (Eq. 1) of the first model and also to simulate the stochastic kinetics with the Gillespie algorithm. In practice, this means that the

focal volume is at thermal equilibrium and the molecular population therein diffuses fast, is homogeneously distributed in space, and is large enough, so that the focal SAS-6 oligomer grows at a constant rate. If any of this assumptions is broken (e.g. if SAS-6 is diffusion-limited due to molecular crowding or, as the reviewer points out, if SAS-6 is spatially restricted by Plk4), it is likely that the quantitative relationship between SAS-6 influx and cartwheel number of the model may change but we expect that the corresponding qualitative behaviour remains the same. In the case where SAS-6 is locally concentrated near the Plk4 focus, as opposed to being uniformly distributed around the centriole(s), both cartwheel assembly and stacking reactions are expected to be faster. This could change how cartwheel numbers and length are distributed along time, but it will not change the fact that stacking inhibits cartwheel assembly in our model. Note, however, that if Plk4 is concentrated at a single site, and SAS-6, in turn, is concentrated at a single site, this means that centriole biogenesis will occur at that site and not, say, at the opposite side of the centriole. It does not follow necessarily that a single centriole will form. This is what our model ensures, under various conditions. We further clarified this point in the text.

In the Models and Methods section we can read (L482-487):
We assume that in the vicinity of a pre-existing mother centriole there is a large enough volume in which the concentrations are isotropic and reaction kinetics can be approximation by mass action, i.e. reaction rates are directly proportional to the concentration of the intervening molecules. We refer to this as the focal volume. This simplification allows us to obtain straightforward analytical solutions and justifies the stochastic simulations described in the next section.

Also in the Discussion we wrote (L433-453):
As mentioned above, Plk4 and STIL cooperate to shuttle SAS-6 onto the centrosome, and eventually organise into a single focus. Under these conditions, if SAS-6 molecules are locally concentrated, this may favour their aggregation and, importantly, long stacks should be able to elongate more easily and inhibit the formation of supernumerary cartwheels. Therefore, it is likely that Plk4, STIL, and SAS-6 focusing facilitates the stacking process. Note, however, that spatial accumulation of these molecules only ensures that centriole biogenesis should happen preferentially in the focus where they are at supercritical concentrations and not elsewhere. It does not ensure per se that one and only one centriole will form at that site without evoking additional control mechanisms, such as stacking mechanism we proposed, […] Altogether, understanding how this model behaves in spatially explicit conditions is an important question to be addressed in the future. We expect that the mechanism we propose should allow centriole assembly control to be even more robust when Plk4, STIL, and SAS-6 focusing is included explicitly.

3)This reviewer assumes that, in reality, the cartwheel stacking ends in the early stage of centriole formation and before genuine centriole elongation, because the outer part of cartwheels is structurally decorated with triplet-microtubules and binding proteins. On the other hand, both SAS-6 total expression level and influx to centrioles are increasing towards late S and G2 phase. This case may correspond to “limited stacking model with high SAS-6 influx”. According to their models and simulations, the risk of forming second cartwheels would be quite high. Is there a way to resolve this discrepancy? In Fig. 6, the authors concluded that “the limited stacking model is OK for cartwheel number control if h is not reached within the time-window of constant SAS-6 influx”, but this condition is unlikely in real centriole formation.

Our simulations indicate that the probability of making supernumerary centrioles can be high if a prescribed maximum length exists and is reached ending elongation within the permissive window. Otherwise, the copy number will be kept under control. Thus, we agree with the judicious comment of the reviewer, however, there is not enough information and for that reason we explored these possibilities theoretically. It is quite possible that, as the reviewer assumes, the cartwheel stacking is completed at early stages before centriole elongation. However, Keller et al. reported an increase in the length of SAS-6 foci in human cells in G2, compared to S-phase, which could be indicative of longer cartwheels. In addition, studies in Chlamydomonas and Spermatozopsis suggest that cartwheel length may vary along the cell cycle. So, it is possible that the cartwheel keeps elongating despite being encircled by centriolar microtubules. In the absence of further definitive data, all plausible assumptions should remain as open possibilities.

4) Related to 3), the stacked cartwheels are inside and settled at the bottom of an elongating centriole, but how could they interfere with formation of second cartwheel assembly at the opposite side of mother centriole (at least ~200 nm away)? There seems to be structural and distance barriers.

The model does not feature spatial constraints. We are assuming that the SAS-6 building blocks are moving freely in the focal volume around the mother centriole and by being sequestered by the elongation of the first cartwheel will decrease its concentration anywhere in that volume, including in the opposite side of the centriole. This is a sink effect that is not unreasonable to be effective at a distance of 200nm or more.

This being said, on L443-L451 we address this potential limitation of the spatial homogeneous model:

Yet it is pertinent to mention spatial effects that we so far neglected. Diffusion rates of macromolecules are expected to decrease with their oligomerisation state, i.e. larger molecules should diffuse more slowly. Therefore, cartwheels should become increasingly less mobile as they elongate, which should diminish their ability to capture freely-diffusing oligomers and relatively higher stacking rates may be necessary to achieve precise number control. The same holds true if there is any physical barrier to SAS-6 diffusion (such as the mother centriole itself). Altogether, understanding how this model behaves in spatially explicit conditions is an important question to be addressed in the future.

5)Although only with a certain range of parameters (e.g. high Ks and low influx, unlimited or limited with enough h), the stacking model actually works. However, it seems to be more vulnerable to guaranteeing one cartwheel assembly compared to the feedback model. This point should be therefore toned down. In a way, the authors nicely verified these models, but this reviewer is wondering how, in reality, the feedback works to inhibit additional cartwheel formation. The authors stated, “not as a specific molecular pathway at play during centriole biogenesis”, but it would be better to discuss how it could work around the pre-existing centriole. The mechanism by which the cartwheel stacking inhibits the assembly of intermediates at other places is not difficult to imagine, but it is not the case for the feedback model.

The feedback model only outperforms the stacking model by a slight margin (2.9% supernumerary cartwheels vs. 2.2%). However, it is important to emphasise that we modelled the feedback using the fastest and simplest possible kinetics: a single reaction with an exponential-distributed waiting time (mode of the distribution is zero such that instantaneous feedback is the most likely event). In contrast, the stacking model was run in the unbiased scenario where the rate of stacking equals that of oligomerisation and dissociation. The performance of the stacking model can be increased substantially by using parameters that favour the stacking kinetically. So, whereas we considered the best possible case for the feedback model, we considered a very conservative case for the stacking model.

Furthermore, the simple feedback model is very unrealistic and the introduction of more realistic features such as multiple reaction steps would deteriorate its performance. Indeed, in signalling pathways with feedback properties, there are usually multiple components that sense a change in the environment, transduce that information, and carry out the final response. The existence of multiple steps and checkpoints would inevitably introduce delays such that the feedback times distribution will have a mode larger than zero as is the case of the gamma distributed times (shown in Figs S8B and S8C). As indicated in the supplementary information, the stacking model outperforms all other multi-step feedback models, which are more representative of actual signaling pathways. We added a comment in this regard (L291-296):

In the feedback model, there is an exponentially-distributed time window of opportunity for the appearance of extra cartwheels. Thus, we observed that the second cartwheel is formed relatively early (at $t \leq 30$) and rarely (2.2% of the simulations), marginally outperforming the stacking model. If $\alpha > 1$, mimicking more realistic multi-component linear feedback pathways, the stacking model is more efficient in averting supernumerary cartwheels (Figs. S8B and S8C).

-Minor points

6)This reviewer would suggest to change the current title “A first-takes-all model of centriole copy number” which sounds too vague. A first-takes-all is applicable to most of reasonable models and theories to explain the principle of centriole duplication, so the authors should specify the title based on their assumptions and models in this study.

We accept the reviewer’s suggestion to make the title more explicit. Note that, in comparison to other models, where multiple centriole primordia (e.g. Plk4-STIL foci) may compete initially — it is not necessarily the first one that “wins”, as in our model. Regardless, we propose the following alteration to better reflect the specificities of our study:

“A first-takes-all model of centriole copy number control based on cartwheel elongation.”

7)The authors should cite "Takao et al., J Cell Biol. 2019", because this is an important paper that conducted mathematical modeling and simulations of centriole duplication.

We agree that this is an important article. We now cite the this article both the introduction and the discussion.

8)Mislabeled in Fig. 3D.

Fixed.

Reviewer #2: In this work, Marco Louro, Monica Bettencourt-Dias and Jorge Carneiro propose a new theoretical model of centriole copy number control. Centrioles are large assemblies crucial for centrosome and cilia formation. Therefore, the number of centrioles per cell must be tighly controlled, but up to now, this mechanism is not yet clearly understood.

In this interesting new model, the authors propose that the stacking of the cartwheel, which is an assembly made of SAS-6 proteins and at the base of centriole formation, would restrict the number of new centrioles to one copy. It's an extremely interesting model that makes sense with a lot of in vivo observations. Unfortunately, I don't have the skills to properly evaluate the simulations. However I have some centriole related questions :

- First of all, I think this article should be more accessible to biologists. I had a hard time reading the article. I guess it's probably not possible to simplify the text but I think that explanatory scheme of the results could help biologists to understand the results/conclusions.

It is always a hard path to navigate between technical accuracy and accessibility to a broad biological audience, particularly in a journal like PLoS Computational Biology. We tried to make the text more accessible by eliminating or explaining some of the more obscure modeller jargon (e.g. “monotonously”, etc.). These are too many to transcribe here but can be found in the markup copy of the manuscript.

- In this model, the stacking of the first cartwheel cause depletion of intermediates, blocking the formation of extra cartwheels. This result works very well with canonical duplication but less well with the formation of centrioles in the case of deuterosomes or blepharoplasts, where dozens of cartwheels are formed. In this case, one can imagine that the concentration of SAS-6 is higher and that it would correspond to the "over-expression" condition tested by the author. In fig. 6, in the limited stacking condition, a 2nd cartwheel forms after the first one. What would be the condition for a dozen cartwheels to form? Is it possible and would they form sequentially? In vivo, at the level of the deuterosomes (or the daughter centriole in the same cells), the assembly seems to be almost synchronous.

We thank the reviewer for the question. The formation of multiple centrioles in deuterosomes or blepharoplasts could indeed correspond to the “overexpression” conditions in our model, as pointed out by the reviewer. In our model, if the influx and stacking rates have a similar value, it is sufficient to ensure the formation of one and only one cartwheel with high probability. To form a dozen centrioles, it would be sufficient to increase the influx rate 10- to 100-fold of the stacking rate. It is important to emphasise however, that in contrast with no variance in the case of a single copy (i.e. all the cells in a population would have one and only one copy) there would be a significant variance in a scenario where the average number of centrioles would be 12.

We added a paragraph where we discuss the consequences of the model outside of canonical centriole duplication and in overexpression conditions:

On L454-L467 we can now read:

Although we discussed our models in the context of duplication of a single pre-existing mother centriole, our results establish how cartwheel number and length depend on SAS-6 availability. One of the predictions of our models is that both the mean and the variance in cartwheel/centriole numbers per cell should increase with higher SAS-6 influx. This is independent of the mode of centriole biogenesis, duplication or de novo. Indeed, it has been observed that both the mean and the variance increase in systems where extra centrioles are produced, such as olfactory neuron progenitors [47], where overproduction occurs via rosettes, and other multicilliated cells, where overproduction occurs at deuterosomes or de novo [48–51]. In all these cases, the extra centrioles are associated with Plk4 and STIL overexpression, which could lead to an increase in centrosomal SAS-6 influx. Therefore, our putative stacking mechanism is in good agreement with these statistics of centriole numbers. Conversely, our model would fail to explain an increase in the mean centriole number without an increase in variance but, to our knowledge, this has never been observed.

-Line 370 "Our models predict that formation of one and only one cartwheel should be robust to random fluctuations in SAS-6 expression within a cell population". It is a very interesting point. Can the authors develop more ? At what concentration could the system no longer control the number of cartwheels? 10 times more, 100, 1000?

We accepted the suggestion of the reviewer and refer to this point specifically. In the discussion of the revised manuscript we wrote (L318-320):

Specifically, the range of σ values that produces a single cartwheel copy in simulations indicates that the control mechanism may be robust and insensitive to broadly distributed concentrations per cell of the regulators of the SAS-6 influx.

-The cartwheel is not only made of rings but also of spokes, probably made of coiled coil domains from different SAS-6 rings. Could this type of organization influence this model? Similarly, the cartwheel is not linear but made of repeating layers. Can the authors comment on this point?

As we understand the comment, it is based on recent discoveries by Nazarov et al. and Klena et al., as well as the 2017 paper by Guichard et al, which show that in the cartwheels of some species, two consecutive rings are connected, which can further organise into higher-order doublets or triplets of double rings.

If so, we would like to recall that we assumed that the "minimal cartwheel" is composed of a single ring. If the "minimal cartwheel" is a ring doublet, or a multiple thereof, we expect that our model should be even more robust to the formation of supernumerary cartwheels, but it may also be likelier that cartwheel assembly fails.

We added a brief discussion of the findings in the aforementioned articles (L378-L386):

Interestingly, several studies have proposed that SAS-6 homodimers in adjacent cartwheel rings interact via their coiled-coils, forming ring doublets and, possibly, even higher-order assemblies in some species [29,40,41]. Thus, it could be that the "minimal cartwheel" is not a single ring, but a ring doublet or a multiple

thereof. This scenario can be readily included in our models by assuming that the formation of a ring doublet rather than a single ring, for instance, is irreversible. In these conditions, our hypothetical stacking mechanism should be even more robust in prevent assembly of extra cartwheels, because forming a new one would require eighteen or more SAS-6 homo dimers to join, instead of nine.

-The authors show that stacking causes depletion of intermediates and inhibits the formation of supernumerary cartwheels. Could this model therefore be applied to the de novo centriole formation ? In the case where there is zero centriole/cartwheel in the cell, a large number of centrioles/cartwheel assemble in the cell and not at the restricted level of a centrosome. Can this model be applied here?

The principle underlying the model could be applied to *de novo* centriole formation. We defined the focal volume as a space around pre-existing centrioles in reference to centriole duplication, but the volume could be extended to the whole cell. Under these conditions, however, the SAS-6 dimer diffusion would be an important player and one would have to make a spatially extended version of the model.

- What do fluctuations in the length of cartwheels mean for the authors? Does this mean that the final length is variable or does the length fluctuate during stacking, such as the dynamic instability of microtubules?

In general, we refer to variation in the final length of the cartwheel after centriole biogenesis is completed in a cell population, which corresponds to the multiple independent simulations under the same parameter regime. In the simulations, cartwheel length can only increase by stacking in the irreversible ring assembly model but can also decrease in the reversible stacking model. In both cases the final length corresponds to the value when the simulation terminates corresponding to the permissive time window in the real cell. In the results section we introduced a clarification (L138-143):

The independent simulations run for each parameter set represent the time courses of the reactions in an independent focal volume around pre-existing mother centriole. Due to the stochastic kinetics, the number and length of Ci_* stacks at the end of the simulations are variable corresponding to the numbers and lengths of the cartwheels across a cell population.

Minor points

-Fig3, the letter of panel “C” should be replace by “D”

Fixed.

-Line 323: There is an extra “the” in this sentence: “some fraction of the this SAS-6”

Fixed.
