# Supplementary material for: A first-takes-all model of centriole copy number control based on cartwheel elongation
Source: PLoS Comput Biol. 2021 May 10;17(5):e1008359. doi: 10.1371/journal.pcbi.1008359 (PMC8136855; doi:10.1371/journal.pcbi.1008359)
Supplement: Attachment — Submitted filename: ReplyToTheReviewer1.pdf [file pcbi.1008359.s011.pdf]

Response to Reviewer 1

Reviewer #1: This reviewer would agree that the authors significantly modified the manuscript following the comments from reviewers. Although no major change was made in the model and simulation data, the premise of the model was totally altered as described below (now in the introduction).

This limitation notwithstanding, the focused accumulation explains naturally the prevention of ectopic centrioles by the maintenance of the key components concentrations below critical values elsewhere in the cell and around the mother centriole. However, these (qualitative or quantitative) proposals fail to explain how the formation of supernumerary centrioles is avoided at the foci, where Plk4, STIL and the other components accumulate at supercritical concentrations. This is the control problem that is solved by the first-takes-all model proposed here.

That is, if this reviewer's interpretation is correct, it is now assumed that the cartwheel competition occurs only at the focus limited by Plk4/STIL and not at other sites around the mother centriole. The authors also assumed here that Plk4/STIL and other components accumulate there at supercritical concentrations. These important premises should be clearly stated in the abstract. Otherwise, it would be impossible to understand what kind of assumptions this model is based on and what is novel in the model, compared with others. Considering all these things, the current abstract is vague and misleading.

We thank reviewer 1 for the relentless effort to help us make the message of our manuscript clearer. We modified the Abstract following the suggestions as follows:

"Here, a novel solution to centriole copy number control is proposed based on the assembly of a centriolar scaffold, the cartwheel. We assume that cartwheel building blocks accumulate around the mother centriole at supercritical concentrations sufficient to assemble one or more cartwheels. Our key postulate is that once the first cartwheel is formed it continues to elongate by stacking the intermediate building blocks that would otherwise form supernumerary cartwheels."

Also, what kind of situation would the authors specifically refer to as supercritical concentration?

Supercritical concentrations must be reached so that centrioles can be formed. As a corollary, every time that a centriole is formed the building blocks and regulators must have reached supercritical concentrations. The questions is then, once such supercritical concentrations are reached why aren't other extra centrioles formed.

To clarify this issue we introduced a brief mention in the abstract as quoted above, and also the following sentence in the introduction (L36-37):

"Supercritical concentrations of these components are necessary to ensure that at least one centriole is formed in either normal or ectopic foci, but are also sufficient to enable formation of extra centrioles (as readily observed in overexpression experiments [55])."

In the current abstract, the authors claim ‘we show that this mechanism may ensure formation of one and only one cartwheel over a wide range of parameter values at physiologically relevant conditions.’ Although ‘over a wide range of parameter values’ is tested by simulations, ‘at physiologically relevant conditions’ seems to be overstatement. This is because, as mentioned above, the model itself is assumed under limited conditions.

We agree with the referee and modified the sentence as follows:

“Using stochastic models and simulations, we show that this mechanism may ensure formation of one and only one cartwheel robustly over a wide range of parameter values.”
